# A Level Set-Based Model for Image Segmentation under Geometric Constraints and Data Approximation

**DOI:** 10.3390/jimaging10010002

**Published:** 2023-12-22

**Authors:** Guzel Khayretdinova, Dominique Apprato, Christian Gout

**Affiliations:** 1 National Institute for Applied Sciences (INSA Rouen), Laboratoire de Mathématiques de l’INSA, 76000 Rouen, France; guzel.khayretdinova@insa-rouen.fr; 2 Tomsk State University of Control Systems and Radioelectronics (TUSUR), Television and Control, 634050 Tomsk, Russia; 3 Université de Pau et des Pays de l’Adour, LMA-UMR 5142, 64000 Pau, France

**Keywords:** energy minimization, level set methods, numerical analysis

## Abstract

In this paper, we propose a new model for image segmentation under geometric constraints. We define the geometric constraints and we give a minimization problem leading to a variational equation. This new model based on a minimal surface makes it possible to consider many different applications from image segmentation to data approximation.

## 1. Introduction

Image segmentation has been heavily studied for more than 40 years in image processing, computer science, mathematics, and from a more general framework in image understanding and computer vision. In [1], the authors present the modeling, techniques, and applications of variational image processing from the theory to the implementation, which constitutes an excellent introduction to image segmentation using variational approaches (including a rigorous study of the mathematical formulations). In [2], the authors give a unified approach of basic computational model reviews for image segmentation, including the Mumford–Shah model (see also [3]), region-based variational active contours, edge-based variational snakes, generalized fast marching method (see also [4]), and active contours. In recent years, deep learning (DL) approaches have been developed (see [5] for a precise and recent state of the art): deep learning approaches have shown qualitatively impressive results but their performance is strongly related to labeled data, and this is, of course, a major drawback on many numerical simulations in case of a lack of labeled data (such that in geosciences, or the specific case of medical images, for instance).

In many applications (geosciences, or even specific medical image processing), the availability of ground truth labels is an important limitation of supervised methods in practice. Another limitation also appears from the excessive cost and time taken to annotate images (in [6,7], the authors estimate that this task requires around 1.5 h of annotation per image in the well-known Cityscapes dataset).

To address this constraint, the study of unsupervised domain adaptation procedures applied to semantic segmentation has been recently conducted in the form of self-training [8]. The addition of geometric constraints makes it possible to improve existing models to obtain good results when acquiring training data is complicated or not possible. In [9], the authors underline that the semi-supervised learning technique is a basic principle which constitutes a strong and efficient solution to consider geometrical constraints in learning semantic segmentation. In [7], the authors propose a multi-modal regularization model applied to self-training procedures in an unsupervised domain linked to semantic segmentation; the introduced regularizer significantly improves self-training methods to various unsupervised domain adaptation benchmarks for semantic segmentation. In [10], the authors propose an enhanced U-Net model with a novel geometric consistency loss for geometry-informed structural component segmentation of post-earthquake buildings, which is of great interest in many applications. In [11], the authors give a new model including a cost term with geometrical constraints during the 2DCNN processing; this term is constructed on a Dice term linked to intensity pairing, a weighted total variation quantity, a piecewise-constant Mumford–Shah-based quantity (see [3] for more details) enforcing intensity homogeneity, and an area penalization. Adding this new cost term with a standard CNN has greatly improved image segmentation results [11].

In [12], the authors introduce a variational approach, in which they combine the approximation from a set of points (like in the model introduced by Zhao et al. [13]) and the curve evolution approach introduced in Caselles et al. [14] under geometric constraints given by the user (points/curves/patches should be taken into account as geometric conditions, as in [15,16,17,18]). This is of great interest in numerical simulations wherein data are incomplete or of insufficient quality. Elsewhere, as stressed in [12], occultation phenomena should appear, making it pertinent to add geometrical constraints in the modeling to guide the image segmentation processing.

In this work, a new model is proposed, improving the segmentation model under geometric constraints, guaranteeing at the same time the convergence (that is not the case in [19]) toward the strong gradients of the image and the approximation of the geometrical data, by giving an energy functional of quadratic type.

In the segmentation process, the geometric constraints (point(s), curve(s), surface patch(es)...) are considered to belong to the searched contour of interest. These conditions are defined manually by the user. The proposed model leads to the minimization of an energy functional, admitting a unique solution, and leading to a variational problem. The introduced model also makes it possible to approximate point cloud datasets, like seafloor or topographic surface approximation (see [20,21] for many applications linked to data approximation). The main focus of this work is about the image segmentation process but we also provide applications to data approximation.

## 2. Modeling

We propose to segment an image $I:\Omega \subset {I\;R}^{3}\to I\;R$. This image is defined after usual regularization (Gaussian, for example): it is well-known that the Gaussian smoothing operator is useful for noise reduction (see Sonka et al. [22]). We consider such a regularization process since we plan to test our method on (potentially noisy) medical images. In case of a large amount of noise, we refer the readers to [23], where the authors introduce a model suitable for segmenting a range of images that have intensity inhomogeneity, noise, and a combination of both.

We propose to segment *I* under geometric constraints. These constraints can be a set of points (as considered in this paper), a set of curves, or a set of surface patches. We consider $\Omega \subset {I\;R}^{3}$ to simplify the problem, but other choices can be made.

We denote by *D* the geometric dataset:

(1) $$D=\left\{a=\left(a_{1},a_{2},a_{3}\right)\in {I\;R}^{3}\right\}\subset \Omega .$$

We denote by $d_{D}$ the distance function defined by

(2) $$d_{D}:\left(x,y,z\right)\in \Omega \to d_{D}\left(x,y,z\right)=\underset{a\in D}{\inf}\left(∥\left(x,y,z\right)-a∥\right)$$

corresponding to the Euclidean distance of the point $\left(x,y,z\right)$ to the set D.

The problem is then the following: we try to find the surface S⊂Ω, *S* being located near the points of maximum gradient of I: it corresponds to the points $\left(x,y,z\right)\in \Omega$ where the usual potential $g\left(∥\nabla I∥\right)=\frac{1}{1+{∥\nabla I∥}^{2}}$ is minimum and considering that the set *D* is close to this set of points (meaning that *S* also approximates the set D).

We now introduce the energy functional E(S):

(3) $$E\left(S\right)=\alpha \int_{S}d_{D}^{2}\left(s\right)ds+\beta \int_{S}g^{2}\left(∥\nabla I∥\right)\left(s\right)ds$$

where α and β are strictly positive, α permits to control the fidelity criterion to the dataset *D*, and β controls the attraction force of *S* linked to the potential $g(∥\nabla I∥).$

To represent the minimal active contour S, we use a level set approach (see Osher and Sethian [24], or Sethian [25]). Minimizing the energy E(S) defined in Equation (3) can be rewritten using the level set approach with $S=\left\{(x,y,z)\in \Omega ;\Phi (x,y,z)=0\right\}$ and Φ the solution of Problem (2):

(4) $$\left|\begin{array}{c} \mathrm{Find}\;\Phi :\Omega \to I\;R\;\mathrm{such}\;\mathrm{that}\\ \forall \xi :\Omega \to I\;R,F\left(\Phi \right)=\min_{\xi }F\left(\xi \right)\\ \mathrm{where}\\ F\left(\xi \right)=\alpha \int_{\Omega }d_{D}^{2}\left(x,y,z\right)\delta \left(\xi \left(x,y,z\right)\right)∥\nabla \xi \left(x,y,z\right)∥dxdydz\\ +\beta \int_{\Omega }g^{2}\left(∥\nabla I∥\right)\delta \left(\xi \left(x,y,z\right)\right)∥\nabla \xi \left(x,y,z\right)∥dxdydz \end{array}\right.$$

where δ is the Dirac in $\xi \left(x,y,z\right)$ (see, for instance, [19], Section 3 for more details).

Problem (4) can be reformulated as

(5) $$\left|\begin{array}{c} \mathrm{Find}\;\Phi :\Omega \to I\;R\;\mathrm{such}\;\mathrm{that}\\ \forall \xi :\Omega \to I\;R,F\left(\Phi \right)=\min_{\xi }F\left(\xi \right)\\ \mathrm{where}\\ F(\xi )=\\ \int_{\Omega }\left(\alpha d_{D}^{2}+\beta g^{2}\left(∥\nabla I∥\right)\right)\delta \left(\xi \right)∥\nabla \xi ∥dxdydz. \end{array}\right.$$

We now suppose that the solution Φ depends on time introducing $t\in ]0,T[)$, and we consider that $\Phi \in W\left(]0,T[;V\right)$ where *V* is a Sobolev space with $V⥀H^{2}\left(\Omega \right)$ to obtain a continuous final contour. The space $W\left(]0,T[;V\right)$ is equipped with its usual scalar product

$${\left(u,v\right)}_{W\left(]0,T[;V\right)}=$$

(6) $$\int_{0}^{T}{\left(u(t),v(t)\right)}_{V}dt+\int_{0}^{T}{\left(\frac{\partial u}{\partial t}\left(t\right),\frac{\partial v}{\partial t}\left(t\right)\right)}_{V^{\prime }}dt.$$

Considering a level set framework [24], the solution S(t) is the zero level at each instant *t*

(7) $$S\left(t\right)=\left\{\left(x,y,z\right)\in \Omega ,\Phi \left(t,x,y,z\right)=0\right\}$$

where the explicit (“mother”) function Φ is the solution of the following evolution problem:

(8) $$\left|\begin{array}{c} \mathrm{Find}\;\Phi \in W\left(]0,T[;V\right)\;\mathrm{such}\;\mathrm{that}\\ J\left(\Phi \right)=\min_{\xi \in W\left(]0,T[;V\right)}J\left(\xi \right)\\ \mathrm{with}\\ J\left(\xi \right)=F\left(\xi \right)+\frac{1}{2}\frac{\partial }{\partial t}\left(\varepsilon \left(t\right){∥\xi \left(t,.\right)∥}_{L^{2}\left(\Omega \right)}^{2}\right),\\ \xi =\xi (.,x,y,z),\mathrm{where}\;F(\xi )\mathrm{is}\;\mathrm{defined}\;\mathrm{in}\;(5)\;\mathrm{at}\;\mathrm{each}\\ \mathrm{instant}\;t,\mathrm{and}\;\mathrm{where}\;\\ \varepsilon \left(t\right)>0,\Phi \left(0,\cdot \right)=\Phi_{0}\in L^{2}\left(\Omega \right)\;(\mathrm{initial}\;\mathrm{condition}). \end{array}\right.$$

The parameter ε introduced in (8) makes it possible to control the variation on time of the energy in space of the solution Φ of (8).

**Figure 1:**
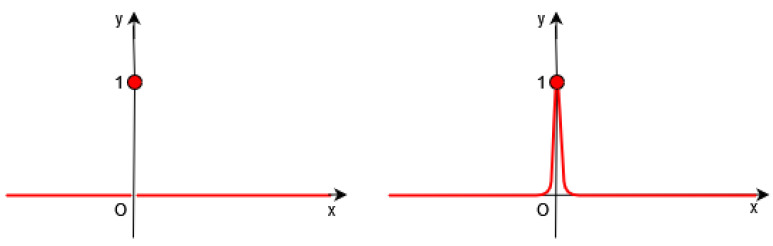
**Left**: Dirac distribution δ (in red). **Right**: In Gout et al.’s work [19], the authors introduce a regularized function $\delta_{\gamma }$ of δ.

**Figure 2:**
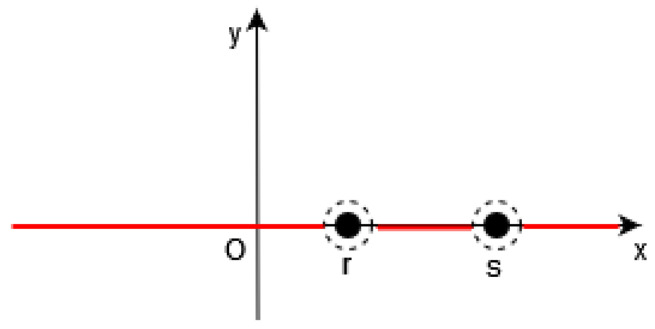
In 1D, in [19], the function $\delta_{\gamma }\left(\Phi \left(t,x,y,z\right)\right)$ (in red) is equal to 0 outside a neighborhood of (x,y,z) such that Φ(t,x,y,z))=0, i.e., outside the points $r,s\in S=\left\{x,\Phi (x)=0\right\}$.

**Figure 3:**
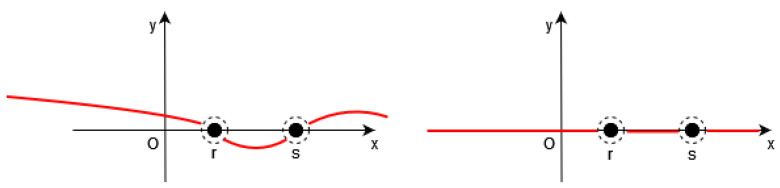
**Left**: the function Φ(t,x,y,z) (in red) is close to a constant between the points of *S*. **Right**: Thus, we have $\nabla \Phi \left(t,x,y,z\right)≅0$ (in red) outside the neighborhood of *S*.

Of course, it is necessary to approximate the Dirac δ by a continuous function in the functional *F* (see Figure 1, Figure 2 and Figure 3).

Here, we propose to replace $\delta_{\gamma }\left(\xi \right)$ by $∥\nabla \xi \left(t,x,y,z\right)∥$; for any *t*, it means that, in first approximation, we suppose that for the solution Φ of (8), we consider that $\delta_{\gamma }\left(\Phi \left(t,x,y,z\right)\right)$ is close to $∥\nabla \Phi \left(t,x,y,z\right)∥$ outside a neighborhood of the zero level S=S(t) of $\Phi \left(t,x,y,z\right)$.

This choice makes it possible to link the behavior of the solution Φ of (8) with weak variation zones of the image *I* (zones where the values of the pixels are almost constant, and thus the gradients are close to zero), and this is performed outside the large variation zones of *I*, that is to say, outside a neighborhood of S=S(t) approximating the set of large variation of *I*.

We also recall that, like in Gout et al.’s work [19], the term $\frac{1}{2}\frac{\partial }{\partial t}\left(\varepsilon \left(t\right){∥\nabla \xi \left(x,y,z\right)∥}_{L^{2}}^{2}\right)$ makes it possible to control the variation in time of the energy in space of the solution of Problem (8). We also state that ε(t)=ε>0 for any $t\in ]0,T[.$ This modeling ensures a simultaneous minimization of both *d* and *g*.

Moreover, a rescaling can be performed by replacing δ with $∥\nabla \xi \left(x,y,z\right)∥$, as performed in Khayretdinova et al.’s work [12]: this rescaling makes it possible to apply the motion to all level sets.

Finally, we obtain the following non-linear energy minimization of a convex functional on the Hilbert space $W\left(]0,T[;V\right):$

(9) $$\left|\begin{array}{c} \mathrm{Find}\;\Phi \in W\left(]0,T[;V\right)\;\mathrm{such}\;\mathrm{that}\\ \tilde{J}\left(\Phi \right)=\min_{\xi \in W\left(]0,T[;V\right)}\tilde{J}\left(\xi \right)\\ \mathrm{with}\\ \tilde{J}\left(\xi \right)=\frac{\varepsilon }{2}\frac{\partial }{\partial t}\left(\int_{\Omega }\xi^{2}\left(t,x,y,z\right)dxdydz\right)\\ +\int_{\Omega }h\left(x,y,z\right){∥\nabla \xi \left(t,x,y,z\right)∥}^{2}dxdydz\\ where\\ h\left(x,y,z\right)=\alpha d_{D}^{2}\left(x,y,z\right)+\beta g^{2}\left(∥\nabla I(x,y,z)∥\right),\\ \alpha >0,\beta >0,\varepsilon >0\;\mathrm{and}\;\Phi \left(0,\cdot \right)=\Phi_{0}\in L^{2}\left(\Omega \right). \end{array}\right.$$

## 3. Main Results

First, we give the variational formulation of our minimization problem. We then give a result about the minimization problem (the convexity comes from that ε>0 and h(x,y,z)>0):

**Theorem 1.**  *Problem (9) is a non-linear energy minimization problem of the convex functional $\tilde{J}$ introduced in Equation (9) on the Hilbert space $W\left(]0,T[;V\right)$ with $V=H^{2}\;\left(\Omega \right)$.*

The uniqueness of the solution Φ comes from this theorem, and we can obtain the following variational formulation (using differential calculus—Gâteaux derivatives—and functional analysis tools): for any $\xi \in W\left(]0,T[;V\right),$∀v∈V, and ∀t∈]0,T[

(10) $$\begin{array}{cc} \tilde{J^{\prime }}\left(\xi \right).v= & \varepsilon \int_{\Omega }\frac{\partial \xi }{\partial t}vdxdydz\\ \; & +2\int_{\Omega }hv\nabla \left(\xi (t,x,y,z)\right)\cdot \nabla \left(v(t,x,y,z)\right)dxdydz \end{array}$$

where $\nabla \left(\right)\cdot \nabla \left(\right)$ is the Euclidean scalar product in ${I\;R}^{3}$ of two gradients.

If we consider that Φ is the solution of Equation (9), this leads to the following theorem:

**Theorem 2.**  *Problem (9) is equivalent to the following problem*

(11) $$\left|\begin{array}{c} Find\;\Phi \in W\left(]0,T[;V\right)\\ \;such\;that\;for\;any\;v\in W\left(]0,T[;V\right),\;and\;\forall t\in ]0,T[\\ \tilde{J^{\prime }}\left(\Phi \right).v=0\\ with\;\Phi \left(0,.,.,.\right)=\Phi_{0}\in L^{2}\left(\Omega \right). \end{array}\right.$$

We can rewrite problem (11) as follows.

Problem (9) is equivalent to the following variational problem:

(12) $$\left|\begin{array}{c} \mathrm{Find}\;\Phi \in W\left(]0,T[;V\right)\;\mathrm{such}\;\mathrm{that}\;\mathrm{for}\;\mathrm{any}\;v\in W\left(]0,T[;V\right)\\ \;\mathrm{and}\;\forall t\in ]0,T[:\\ \varepsilon \int_{\Omega }\frac{\partial \Phi }{\partial t}vdxdydz+a\left(\Phi ,v\right)=0\\ \;\mathrm{and}\;\Phi \left(0,\cdot \right)=\Phi_{0}\in L^{2}\left(\Omega \right), \end{array}\right.$$

where the bilinear form a(·,·) on V×V is defined as

(13) $$a\left(u,v\right)=2\int_{\Omega }h\left(x,y,z\right)\left[\frac{\partial u}{\partial x}\frac{\partial v}{\partial x}+\frac{\partial u}{\partial y}\frac{\partial v}{\partial y}+\frac{\partial u}{\partial z}\frac{\partial v}{\partial z}\right]dxdydz$$

with $h\left(x,y,z\right)=\alpha d_{D}^{2}\left(x,y,z\right)+\beta g^{2}\left(∥\nabla I(x,y,z)∥\right).$

Let us note that the bilinear form defined in Equation (13) is symmetric, continuous on V×V since h(x,y,z) is positive, and superiorly bounded by $\left(\alpha \times d_{H}\left(\Omega ,D\right)+\beta \right)$ where $d_{H}$ represents the Hausdorff distance.

## 4. Numerical Examples

### 4.1. Discretization of the Variational Problem

The discretization is performed using finite differences in time and finite elements in space. We have chosen the $C^{1}$ Bogner Fox Schmidt rectangle as generic finite elements (see Ciarlet [26]). We approximate a(·,·) using a quadrature formula (using the nodes of a voxel grid of image I).

#### 4.1.1. Discretization on Time

We divide $]0,T[$ into *m* subintervals of equal lengths δt: we introduce the steps $t_{m},\forall m\in \left\{1,2,...,\frac{T}{\delta t}\right\},$ and we have

$$t_{m}=m\delta t.$$

We then use a classical finite difference scheme to approximate $\frac{\partial \Phi }{\partial t}\left(t,x,y,z\right)$:

(14) $$\left\{\begin{array}{c} \forall m\in \left\{1,2,...,\frac{T}{\delta t}\right\},\\ \frac{\partial \Phi }{\partial t}\left(t_{m},x,y,z\right)≃\frac{\Phi \left(t_{m},x,y,z\right)-\Phi \left(t_{m-1}\right)\left(x,y,z\right)}{\partial t}. \end{array}\right.$$

#### 4.1.2. Discretization of the Bilinear Form

The main difficulty in this part is the discretization of the function *h*. This function uses the computation of the distance $d_{D}$ and the computation of the potential g.

We propose to use a quadrature formula: for any measurable function *f* on Ω

(15) $$\int_{\Omega }f\left(x,y,z\right)dxdydz≃\underset{i=1}{\sum^{N}}\lambda_{i}f\left(x_{i},y_{i},z_{i}\right)$$

where ${\left(x_{i},y_{i},z_{i}\right)}_{i}$ are the nodes of the quadrature formula and ${\left(\lambda_{i}\right)}_{i}$ the corresponding weights.

Considering that the function *h* is applied on the values of the image *I* (via the function *g*), we choose to take for the nodes ${\left(x_{i},y_{i},z_{i}\right)}_{i}$ the centroids of the voxels of the image I. This makes it possible to compute

$$g^{2}\left(∥\nabla I(x_{i},y_{i},z_{i})∥\right)=\frac{1}{{\left(1+{∥\nabla I(x_{i},y_{i},z_{i})∥}^{2}\right)}^{2}}$$

after having discretized the term $\left(∥\nabla I∥\right)$ using finite differences.

Moreover, the choice of the weights ${\left(\lambda_{i}\right)}_{i}$ should be made such that high degrees polynomials satisfy the quadrature Formula (15), to have a numerical integration error which is consistent with the approximation error of the space $V=H^{2}\;\left(\Omega \right)$ by the finite element space $V_{h}$ we will introduce in the following subsection.

Therefore, at this stage, we replace the bilinear form a(·,·) by

(16) $$\left\{\begin{array}{c} \forall u,v\in V,\\ \tilde{a}\left(u,v\right)=2\sum_{i=1}^{N}\lambda_{i}h\left(x_{i},y_{i},z_{i}\right)\left(\frac{\partial u}{\partial x}\frac{\partial v}{\partial x}+\frac{\partial u}{\partial y}\frac{\partial v}{\partial y}+\frac{\partial u}{\partial z}\frac{\partial v}{\partial z}\right)\left(x_{i},y_{i},z_{i}\right) \end{array}.\right.$$

#### 4.1.3. Discretization on Time and Space

Following the discretization in time and the discretization of a(·,·), the Problem (12) is approximated by the following: for any $m\in \left\{1,2,...,\frac{T}{\delta t}\right\},$ noting $\Phi^{m}=\Phi \left(t_{m}\right),$

(17) $$\left\{\begin{array}{c} \mathrm{Find}\;\Phi \in W\left(]0,T[;V\right)\;\mathrm{such}\;\mathrm{that},\;\forall m\in \left\{1,2,...,\frac{T}{\delta t}\right\},\forall v\in V,\\ \varepsilon \int_{\Omega }\Phi^{m}\left(x,y,z\right)v\left(x,y,z\right)dxdydz+\delta t\;\tilde{a}\left(\Phi^{m},v\right)\\ =\varepsilon \int_{\Omega }\Phi^{m-1}\left(x,y,z\right)v\left(x,y,z\right)dxdydz \end{array}\right.$$

where $\tilde{a}\left(\cdot ,\cdot \right)$ is defined in (16), with $\Phi^{0}=\Phi_{0}\in L^{2}\left(\Omega \right)$ (initial condition).

Now, we have to discretize Problem (17) on space; thus, we have to approximate $V=H^{2}\;\left(\Omega \right)$ by a finite element space $V_{h}\subset V.$

We consider a meshing (Bogner Fox Schmit, for example, see [26] for more details) corresponding to the voxel grid of the image *I*.

We then consider a finite element space $V_{h}:V_{h}$ is of finite dimension ($\dim V_{h}=M\left(h\right)=M$), and $V_{h}\subset V.$ Let ${\left(\varphi_{j}\right)}_{j=1,...,M}$ be a basis of $V_{h}.$

We state

(18) $$\left\{\begin{array}{c} \forall m=1,...,\frac{T}{\delta t},\\ \Phi^{m}=\sum_{i=1}^{M}\alpha_{j}^{m}\varphi_{j}\\ \mathrm{and}\;\alpha^{m}={\left(\alpha_{j}^{m}\right)}_{j=1,...,M}. \end{array}\right.$$

As usual, we then take $v=\varphi_{l}\in V_{h}\subset V,l=1,...,M,$ and using (18), we then deduce that Problem (17) is approximated by

(19) $$\left\{\begin{array}{c} \forall m\in \left\{1,2,...,\frac{T}{\delta t}\right\},\mathrm{find}\;\alpha^{m}={\left(\alpha_{j}^{m}\right)}_{j=1,...,M}.\;\mathrm{such}\;\mathrm{that},\forall l=1,...,M,\\ \varepsilon \sum_{j=1}^{M}\alpha_{j}^{m}\int_{\Omega }\varphi_{j}\left(x,y,z\right)\varphi_{l}\left(x,y,z\right)dxdydz\\ +\delta t\sum_{j=1}^{M}\alpha_{j}^{m}\tilde{a}\left(\varphi_{j},\varphi_{l}\right)\\ =\varepsilon \int_{\Omega }\Phi^{m-1}\left(x,y,z\right)\varphi_{l}\left(x,y,z\right)dxdydz\\ \mathrm{with}\;\Phi^{0}=\Phi_{0}\in L^{2}\left(\Omega \right)\;(\mathrm{initial}\;\mathrm{condition}). \end{array}\right.$$

Let us note that the initial guess $\Phi^{0}=\Phi_{0}$ is chosen in order to obtain

$$\Phi_{0}\in C^{k}\left(\Omega \right)\subset V\subset L^{2}\left(\Omega \right),k\in {I\;N}^{*},$$

so, this initial condition is regular enough to define its interpolation $\Phi_{0}^{h}\in V_{h}$ such that

$$\Phi_{0}≃\Phi_{0}^{h}=\sum_{j=1}^{M}\alpha_{j}^{0}\varphi_{j}\in V_{h}$$

where $\alpha^{0}={\left(\alpha_{j}^{0}\right)}_{j=1,...,M}$ are the degrees of freedom of $\Phi_{0}$ in $V_{h}.$

We now write

(20) $$\left\{\begin{array}{c} \forall m\in \left\{1,2,...,\frac{T}{\delta t}\right\},\mathrm{find}\;\alpha^{m}={\left(\alpha_{j}^{m}\right)}_{j=1,...,M}.\;\mathrm{such}\;\mathrm{that},\;\forall l=1,...,M,\\ \varepsilon \sum_{j=1}^{M}\alpha_{j}^{m}\int_{\Omega }\varphi_{j}\left(x,y,z\right)\varphi_{l}\left(x,y,z\right)dxdydz\\ +\delta t\sum_{j=1}^{M}\alpha_{j}^{m}\tilde{a}\left(\varphi_{j},\varphi_{l}\right)\\ =\varepsilon \sum_{j=1}^{M}\alpha_{j}^{m-1}\int_{\Omega }\varphi_{j}\left(x,y,z\right)\varphi_{l}\left(x,y,z\right)dxdydz\\ \mathrm{with}\;\alpha^{0}={\left(\alpha_{j}^{0}\right)}_{j=1,...,M}\in {I\;R}^{M}. \end{array}\right.$$

Problem (18) can be written as a linear system, and we first write

(21) $$\left\{\begin{array}{c} R=\left(R_{jl}\right)\in M_{M,M}\left(I\;R\right)\;\mathrm{with}\\ \forall j,l=1,...,M,\\ R_{jl}=\varepsilon \int_{\Omega }\varphi_{j}\left(x,y,z\right)\varphi_{l}\left(x,y,z\right)dxdydz+\delta t\;\tilde{a}\left(\varphi_{j},\varphi_{l}\right)\\ \mathrm{and}\\ L^{m-1}=\varepsilon \left(L_{l}^{m-1}\right)\in M_{M,1}\left(I\;R\right)\;\mathrm{with}\;\forall l=1,...,M,\\ L_{l}^{m-1}=\varepsilon \sum_{j=1}^{M}\alpha_{j}^{m-1}\int_{\Omega }\varphi_{j}\left(x,y,z\right)\varphi_{l}\left(x,y,z\right)dxdydz. \end{array}\right.$$

and then, we obtain the following linear system with (20) and (21):

(22) $$\left\{\begin{array}{c} \forall m\in \left\{1,2,...,\frac{T}{\delta t}\right\},\\ \mathrm{find}\;\alpha^{m}\in {I\;R}^{M},\;\mathrm{such}\;\mathrm{that}\;\\ R\alpha^{m}{=L}^{m-1}\\ \mathrm{with}\;\alpha^{0}\in {I\;R}^{M}. \end{array}\right.$$

### 4.2. Image Segmentation

To illustrate our approach, we choose to consider the BraTS Dataset [27]. We take an analogous process as we did in [12]: we take 274 MR scans, each with four MIR sequences. The training data have the size 240 × 240 × 155 pixels, and we obtain the (manual) segmentation labels for different brain tumors. We trained the deep network using 79 training data, and we set the initial learning rate as $10^{-4}$ and multiplied this by 0.5 after every 20 epochs.

**Figure 4:**
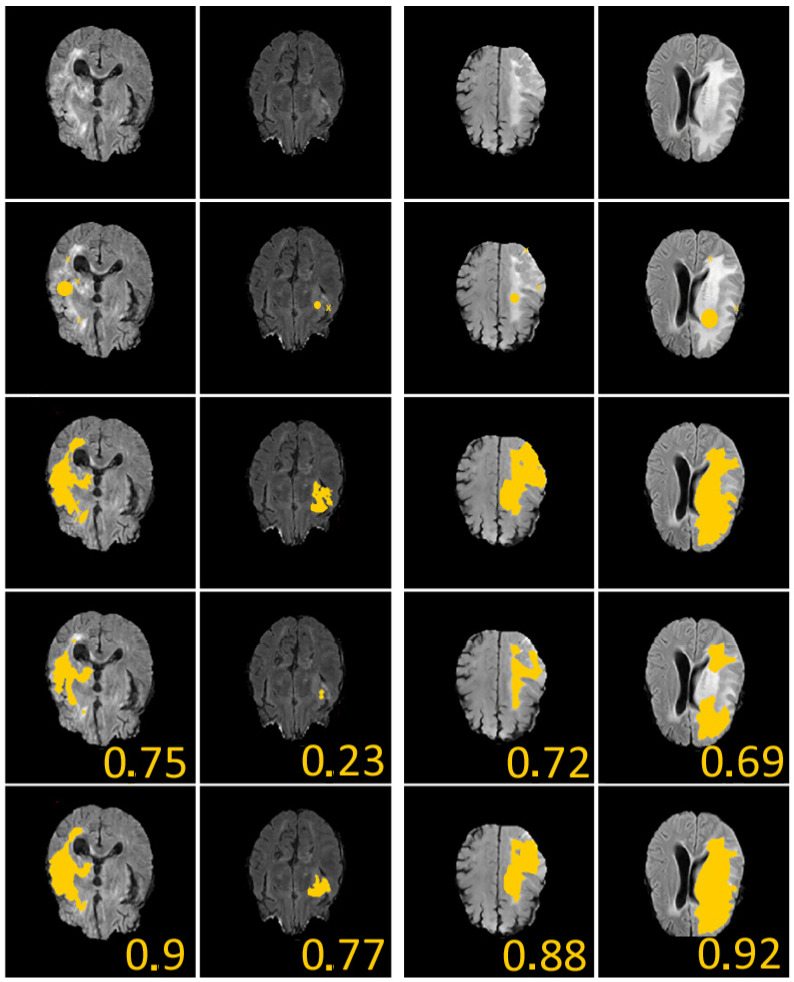
We give 4 examples from the Brats dataset [27] with comparisons between our method and U-Nets. First line: considered images. Second line: initial guess (yellow crosses represent geometrical conditions (set of point(s)), and yellow discus is zero level of initial condition). Third line: ground-truth labels. Fourth line: segmentation obtained using supervised U-Nets [28]. Fifth line: segmentation obtained with our algorithm. In all examples, we considered α=1, β=1, δt=0.3, and δx=0.1. The given values represent the Dice score. These results illustrate the efficiency of our proposed approach.

To define the geometric constraints, we choose two to three points given by the user and located near the boundary of the part we want to segment. Let us note that the choice of the geometric constraints (two or three points in all our tests) modifies the dice score by less than 6% on the 150 different tests we have conducted on the examples of the second and fourth column of Figure 4, but it reaches 20% on several cases, for example, in the first and third columns of Figure 4. An explanation for this is that, in these images, the contours are rather blurred/noisy. Regarding the sensitivity of the model, we can say that the choice of geometric conditions logically impacts the final result, especially in very noisy areas.

We recall that a high Dice value or a small Hd value represent a high-quality segmentation result. In Table 1, we notice that the computational time is acceptable with our method (Chan–Vese being the fastest). The quality of the segmentation result is analogous: our algorithm and the one of [12] are slightly better. Of course, we have to take into account that our approach is not user-free: the user has to define the geometric conditions (with the mouse), and several parameters have to be defined (time step, space step, α, β, and the initial condition for the level set approach which is a cone in all cases.) Our approach has the benefits of topological independence given by the level set method [24]. Our method is fast and very useful when not working with many labeled datasets, or when having blurred data or missing data on the image.

**Remark 1.**  *The main difference between the model presented in this paper and the model given in the work of Gout and Le Guyader [16] is the assurance that we (here) minimize both d and g simultaneously, while the energy to be minimized in [16,19] does not guarantee this point (where the minimization concerns the product d×g). In [12], the advantage is the initial guess (generated from the geometric conditions), but the approximation method is less efficient than the one presented in this paper.*

**Figure 5:**
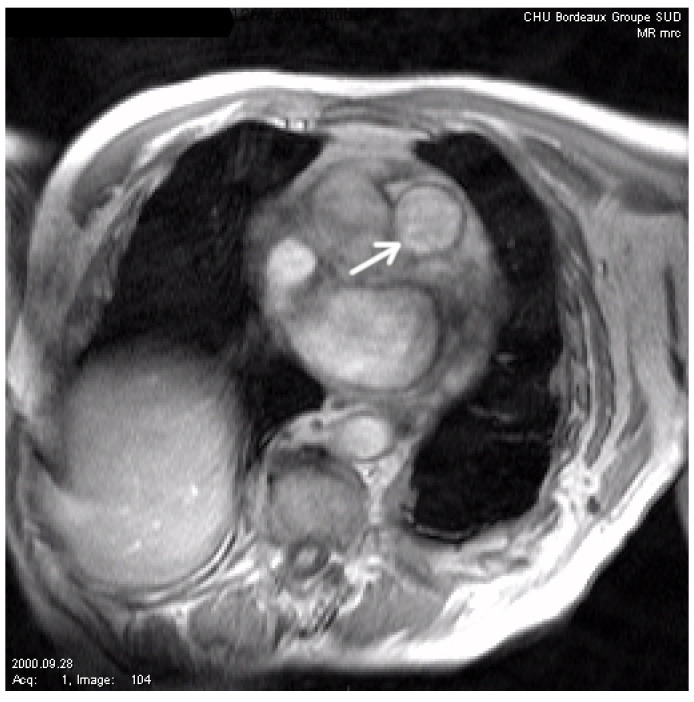
Initial image. The arrow shows the vessel to be segmented (main pulmonary artery).

**Figure 6:**
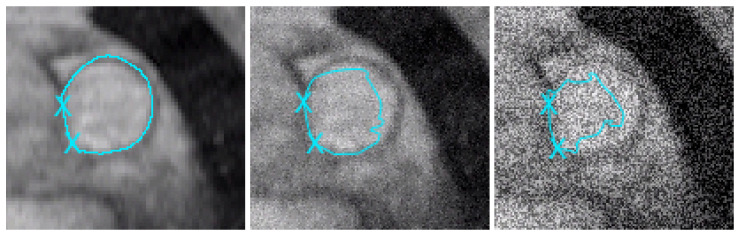
Studied zone around the main pulmonary artery. We use the model proposed in this paper. **Left**: the MPA is perfectly segmented on the initial image (we have considered 2 points as geometric conditions). We obtain equivalent results until adding 40% of noise. **Middle**: after adding 50% of noise on the initial image, the geometric conditions are efficient, but in the right part of the artery, the segmentation contour is (logically) distorted by the noise. **Right**: after adding 200% of noise, the result is of course worse (except near the geometric conditions).

**Figure 7:**
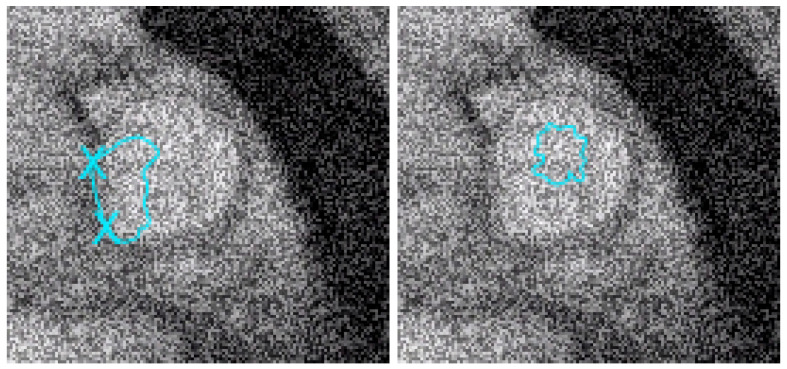
**Left**: with the same geometric conditions as in Figure 6 and on the image with the addition of 200% of noise, we test the algorithm given in [12]. We can see that the result is equivalent to the one of our approach near the 2 points to be interpolated but worse than with our algorithm in other zones. **Right**: the geodesic active contours (without interpolation conditions) do not give a good result (it is well-known that they are sensitive to noise).

To test the robustness of our algorithm, we now present several comparisons on noisy images. In Figure 5, we show the considered image (courtesy of CHU Bordeaux, this image represents a slice set perpendicularly to the main pulmonary artery axis). We then artificially add noise to this image, and we compare our algorithm with the one of [12] and the classic geodesic active contour without geometric conditions (in Figure 6 and Figure 7). We can see that our method gives the best results, although it remains sensitive to noise. It is, of course, possible to improve the modeling in the case of applications to image segmentation of noisy images by adding, for instance, the gradient vector flow in the modeling, as stressed in [30].

### 4.3. Data Approximation

The main objective of the proposed modeling is image segmentation under geometric constraints, but it is also of interest to underline that our proposed modeling is efficient for data approximation from a large amount of data. Data approximation remains an important research field. For example, ocean mapping to obtain a complete map of the Earth’s seabed is a main objective of the next years: this is a crucial point to better understand many environmental challenges from ocean circulation and climate models to tsunami forecasting, cable routing, sediment transportation, renewable energy production, rising of a submarine volcano becoming a new island on a hot spot (like in Hawai’i), etc.

Projects like TOPEX (https://topex.ucsd.edu/ (accessed on 21 December 2023)) (Scripps Institution of Oceanography, UC San Diego) or more recently GEBCO (https://www.gebco.net/data_and_products (accessed on 21 December 2023)) (International Hydrographic Organization and the Intergovernmental Oceanographic Commission of UNESCO) focus on Earth mapping (especially seafloor surfaces). Another current project is Seabed 2030 (of the Nippon Foundation and the General Bathymetric Chart of the Oceans nonprofit organization), the goal being to map the entire seafloor by 2030 (we are currently at 21%, we were at only 6% in 2017 [31]). Elsewhere, it is of course of interest to propose a surface approximation method from topographic data (from Earth, or other planets like Mars) or lidar/bathymetry data to obtain the value of a surface on every point of a studied domain. Of course, several well-known approaches have been introduced like spline approximation or spline under tension (see [32] for more details), $D^{m}$ spline [20,21,33,34], or kriging [35]. All these methods have drawbacks like presenting oscillations in the case of rapidly varying data (spline functions), or lack of regularity of the obtained approximant (kriging), or difficulties in managing significant amounts of data (approximation using polynomial approximation with a significant CPU cost...).

**Figure 8:**
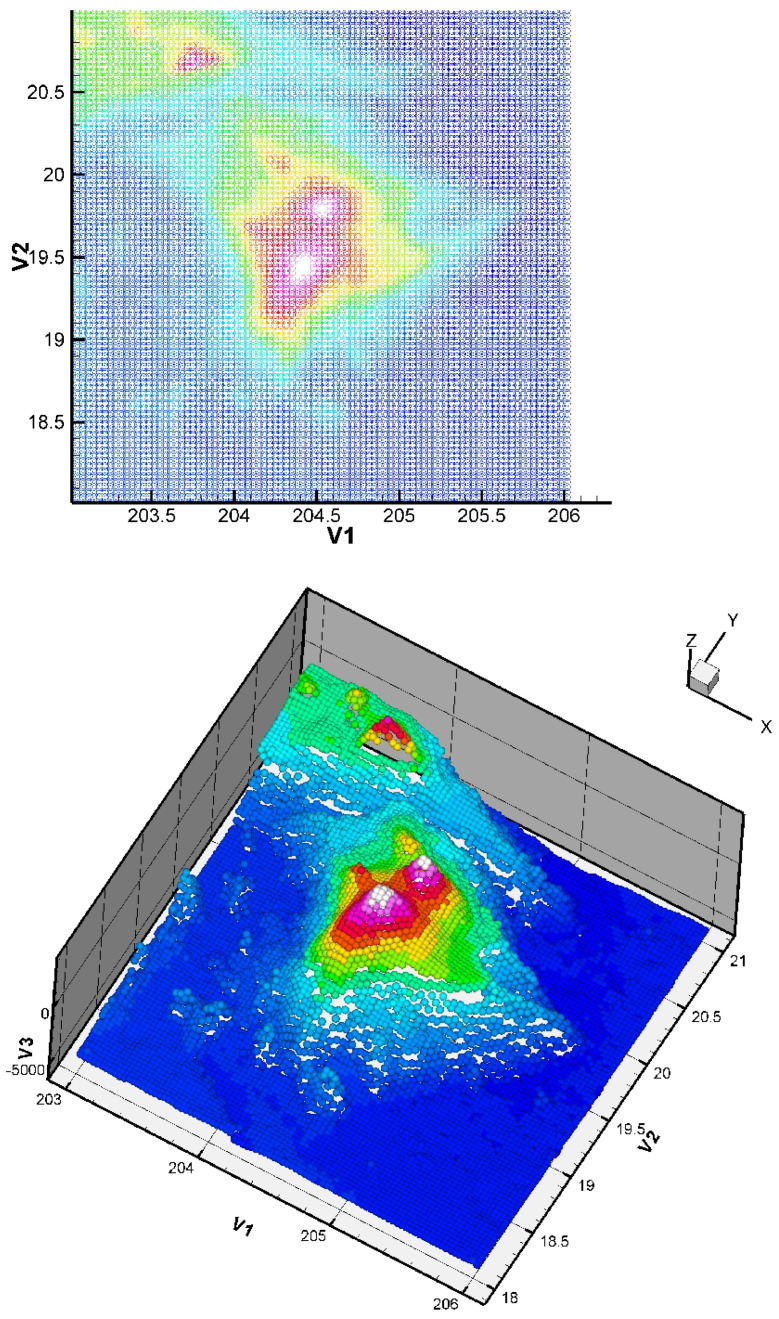
Two-dimensional and three-dimensional views of the dataset: Big Island (Hawaii) zone, 8736 points.

**Figure 9:**
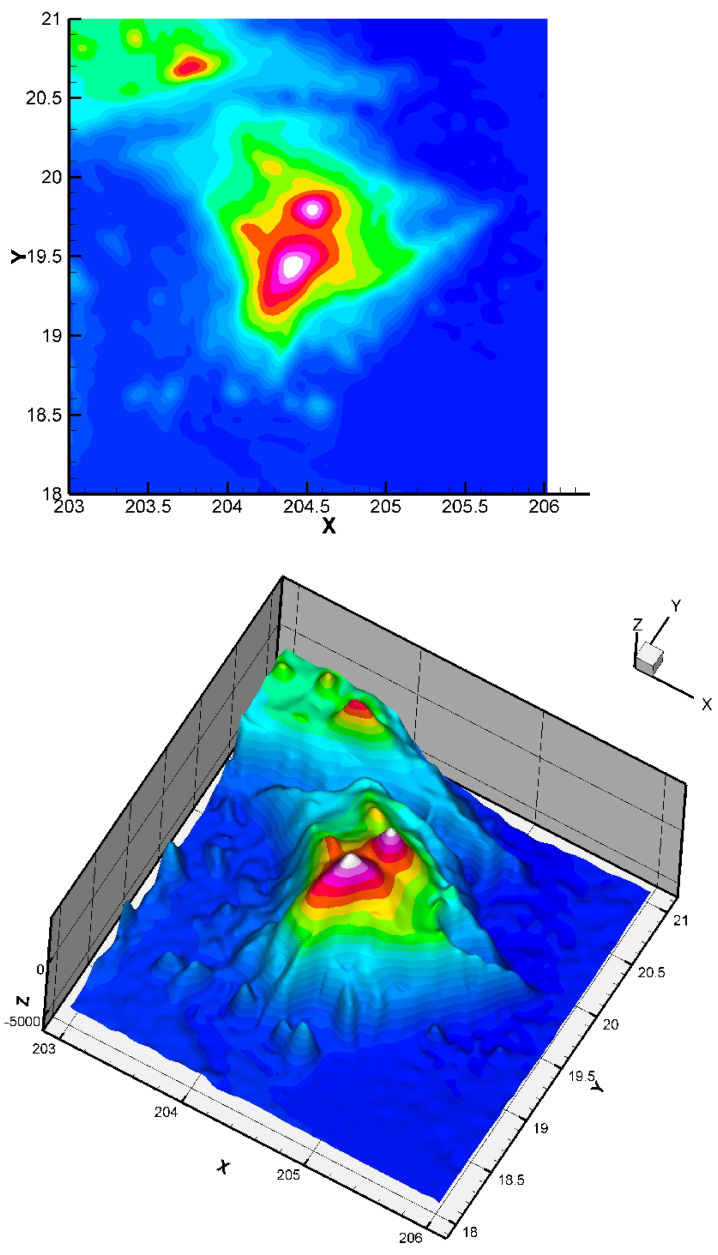
Obtained approximation of the Big Island (Hawai’i) zone using a finite element grid of 400 Bogner Fox Schmidt rectangles (of class $C^{1}$, see [26] for more details). The step δt is equal to 0.3. The quadratic error (23) is equal to $6.8\times 10^{-5}.$ Such quadratic error values are very good in the surface approximation framework, and show that our approach is efficient, even in the case of this rather complex dataset (having large variations). In the global dataset, the maximum error measured is 6%, corresponding to a maximal error of 42 m (the location of this maximum error is logically near the steep valleys).

Here, we consider the energy functional E(S) of Equation (3) with α>0 and β=0. The initial condition has no impact on the quality of the result (but it has a small impact on the CPU time). The considered dataset was obtained from the TOPEX project. The dataset is constituted of 8736 points giving the seafloor surface and topography of Maui and the Big Island, Hawai’i (Figure 8). This zone (around Mauna Kea) is of interest since it is the largest “mountain” on Earth from base to top (from −7000 m to +4207 m, so around 11,207 m in total) and this zone is in permanent evolution (Hawaiian hot point, with active volcanoes like Kilauea, Mauna Loa, Mauna Kea, or the youngest volcano Kama’ehuakanaloa). We give the approximation in Figure 9.

To compute the error rate, we recall the quadratic error formula:

(23) $$Quad\_Err={\left(\frac{\sum_{i=1}^{1500}{\left({\tilde{z}}_{i}-z_{i}\right)}^{2}}{\sum_{i=1}^{1500}z_{i}^{2}}\right)}^{1/2},$$

where $z_{i}$ is the value of the z-data, and ${\tilde{z}}_{i}$ is the value of the z-approximation for the (same) point $\left(x_{i},y_{i}\right)$.

To compute quadratic errors (Table 2), we only consider 7236 points of the dataset (out of 8736) and we compute the error of Equation (23) on the 1500 randomly deleted points.

The best error is obtained by the $D^{m}$-spline operator of [21], and our proposed method here is (rather) equivalent in terms of error, but faster than the $D^{m}$-spline (see Table 3). Kriging is the fastest but the approximation quality is inferior. A drawback of our approach is that it requires an initial condition while it is not necessary when using spline approximation/kriging. The CPU time with our proposed approach can be improved: instead of using finite elements (that have the advantage of guaranteeing a $C^{1}$ regularity of the final surface), it is possible to use the fast sweeping scheme (Gauss–Seidel iterations with alternating orderings) to solve the Eikonal equation and the Euler–Lagrange equations can be computed by gradient descent algorithm, and with finite differences in the discretization.

## 5. Conclusions

We have proposed efficient modeling for both image segmentation under geometric constraints and data approximation.

For image segmentation, the considered constraints are a set of points to interpolate, but other choices can be made (set of curves, surface patches...). The user defines these points with the mouse.

The role of these geometric constraints is multiple: they can contribute to the acceleration of the convergence of the algorithm by having a similar role as the inflation force of the Balloons model [37], or they can be imposed in the model as wells data in geophysics [17]. These geometric conditions are also useful when image data are blurred or are missing (to help the segmentation process).

We insist on the fact that the goal of our variational approach is not to challenge DL approaches when having a significantly large set of labeled datasets, the goal is mainly to use it on specific cases (when not having a labeled dataset, which is the case in several medical applications and segmentation in geophysics).

In all our segmentation examples, we have kept the same values of the coefficients that modulate the relative weight of the data fidelity term (β) and the one associated with the gradient modulus measure (α) can be optimized to improve the results (but it makes the method less user-free, which is why we have chosen α=β=1).

**Figure 10:**
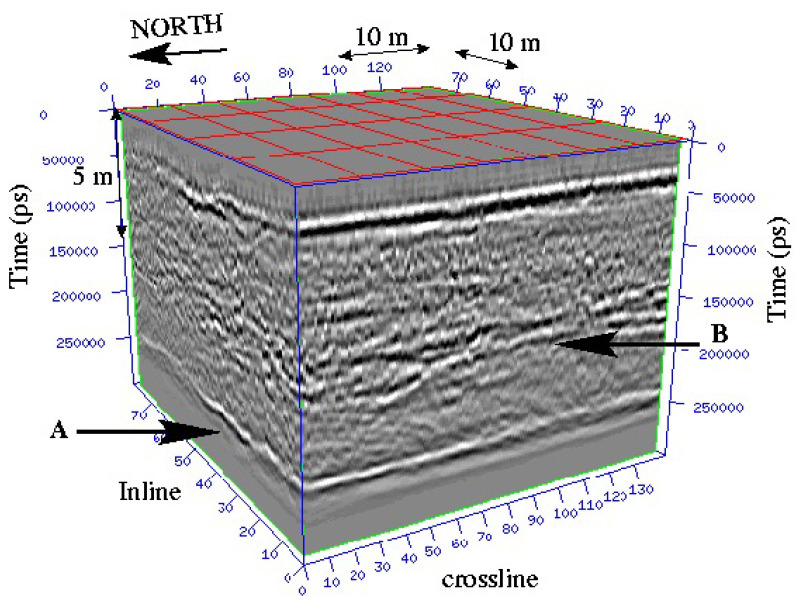
Example of a 3D seismic dataset wherein two continuous reflectors (layer A and layer B) appear.

**Figure 11:**
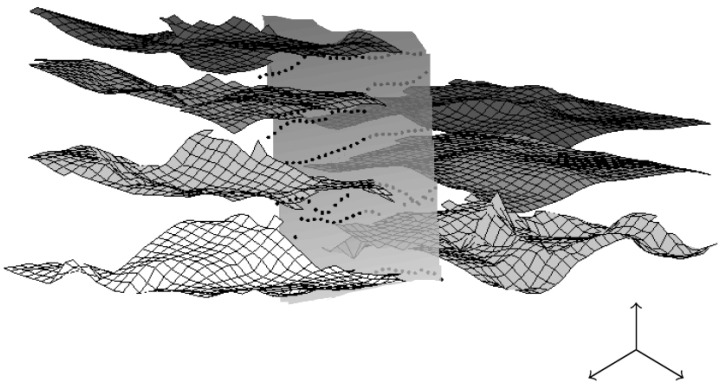
An example of layers and a vertical fault extracted from the complex 3D dataset of Figure 10. Obtaining such visualization requires for a geologist to directly work on the 3D bloc (almost pixel after pixel); we propose to use a segmentation process with geometric constraints to segment one layer after another.

The first numerical results we obtained are very promising. Of course, other tests and comparisons (geophysical datasets like seismic datasets, and 3D datasets in medicine or geosciences...) have to be conducted (work in progress). In Figure 10, we show a 3D geophysical dataset: from this dataset, the goal is to be able to give a visualization of layers and faults like on Figure 11 using a segmentation process; the geometric conditions correspond in this case to wells data, helping the segmentation process to obtain the right layer.

This is a work in progress: a current difficulty we face consists in having a sufficiently good visualization to choose the points (geometric conditions given by the user) inside the 3D bloc; this is unsolved for now (for 3D datasets, we have unsuccessfully tried to link our segmentation process with Paraview [38] and Tecplot [39] so far).

Regarding the parameters, the spatial step is (in general) chosen so that a finite element of the mesh comprises approximately 25 to 100 pixels. Let us note that this leads to more relevant results if we choose a smaller space step (which involves solving a larger linear system). The time step is related to the accuracy of the calculation: less errors will be made between two iterations if we choose a small time step. Values of the order of $10^{-3}$ seem to be suitable in many tests we have conducted and a smaller number of iterations can be given as a stop criterion.

Moreover, in the energy defined in Equation (3), it is possible to consider the following cases to obtain many different applications:

- α>0 and β>0: it corresponds to our proposed segmentation model under geometric conditions.
- α=0 and β>0: it corresponds to a basic segmentation model without geometric conditions.
- α>0 and β=0: it corresponds to data approximation from a finite set of data with potential applications to seafloor surfaces approximation from various kinds of data (ship tracks data in bathymetry, lidar measurements...) or to shape optimization.

**Figure 12:**
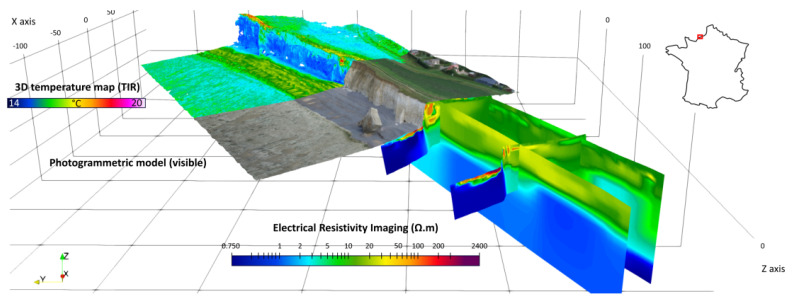
An example of a studied zone in Normandy (Sainte Marguerite cliffs). From different datasets (including acquisition using drones carrying infrared cameras and photogrammetry). The goal is to precisely reconstruct the topography (credits: Defhy3geo project, with Cerema Normandie).

**Figure 13:**
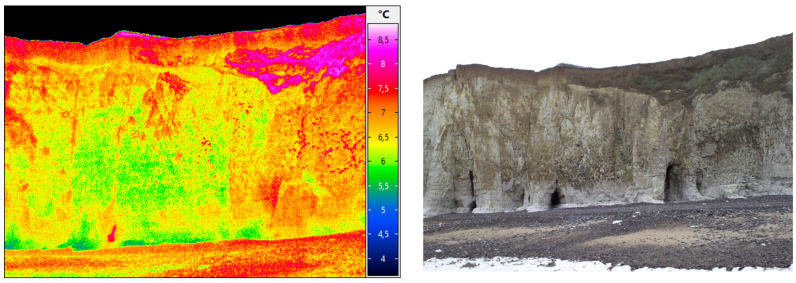
A studied zone with infrared datasets (Vaches noires cliffs, credits: Defhy3geo project, with Cerema Normandie). Approximation of coastal zones is required for many applications like security concerns (cliffs collapsing), or to study the impact of topography on velocity wind fields (Intertwind project).

Another work in progress concerns the data approximation of coastal cliffs in Normandy (France) from topographic datasets and from infrared datasets (see Figure 12 and Figure 13).

This general framework is promising, and the model Equation (3) can be developed, adding new kinds of geometric datasets, such as surface patches, Hermite datasets with tangent planes to given 3D datasets, wind velocity fields approximation from lidar datasets, in applications both linked to image segmentation and data approximation.

## Figures and Tables

**Table 1:** Precision of segmentations models on 1/3 of labeled data from BraTS dataset [27]. The computations were conducted on a Nvidia GeForce RTX 2080 (GPU memory: 11 GB). We compare our method with U-Net, with the image segmentation approach under geometric constraints of [12] (taking equivalent points as initial condition as we did with our method) and with the Chan–Vese segmentation method ([29]) (the initial guess here corresponds to a closed contour located inside the region of interest). We give the results of classical metrics in image segmentation: mean Intersection over Union (mIoU), Dice, Hausdorff distance (Hd), and GPU time for these 4 segmentation methods.

Method	mIoU	Dice	Hd	GPU Time
U-Net [28]	78.3	87.7	43.5	4.45
Chan–Vese [29]	77.6	88.1	41.5	2.02
Khayretdinova et al. [12]	79.6	89.1	39.5	2.12
Our method	79.4	89.1	39.5	2.72

**Table 2:** Error tables: we give the quadratic error (23) between the obtained approximation and the dataset. We give the results for 2 different finite element meshes.

	Mesh	Mesh
**Method**	**20 × 20**	**10 × 10**
Spline [21]	0.0000045	0.00026
Kriging [36]	0.0074	0.0074
Our method	0.000068	0.00092

**Table 3:** Tests are carried out on a 2.7 GHz laptop with an Intel Core i7-7500U CPU @2.70 GHz, 2901 MHz. We give a comparison of the CPU time between the different methods we have tested. We give the results (in seconds) for 2 different finite element meshes. Of course, there is no mesh needed for kriging.

	Mesh	Mesh
**Method**	**20 × 20**	**10 × 10**
Spline (Fortran) [21]	26 s	11
Kriging (C++) [36]	4	4
Our method (C++)	16	9

## Data Availability

Data are contained within the article.
